# 4-Hydroxyphenylacetic Acid Attenuated Inflammation and Edema via Suppressing HIF-1α in Seawater Aspiration-Induced Lung Injury in Rats

**DOI:** 10.3390/ijms150712861

**Published:** 2014-07-21

**Authors:** Zhongyang Liu, Ronggang Xi, Zhiran Zhang, Wangping Li, Yan Liu, Faguang Jin, Xiaobo Wang

**Affiliations:** 1 Department of Pharmacological and Toxicological Research Centre, No. 210 Hospital of Chinese People’s Liberation Army, Dalian 116021, China; E-Mails: lhw@wfmc.edu.cn (Z.L.); yyyes@wfmc.edu.cn (R.X.); wangfei@wfmc.edu.cn (Z.Z.); shifuyan@wfmc.edu.cn (Y.L.); 2 Department of Respiration, Tangdu Hospital, Fourth Military Medical University, Xi’an 710038, China; E-Mail: wangdongrui@gmail.com

**Keywords:** 4-hydroxyphenylacetic acid, seawater drowning, hypoxia-inducible factor 1α, acute lung injury

## Abstract

4-Hydroxyphenylacetic acid (4-HPA) is an active component of Chinese herb *Aster tataricus* which had been widely used in China for the treatment of pulmonary diseases. The aim of this study is to investigate the effect of 4-HPA on seawater aspiration-induced lung injury. Pulmonary inflammation and edema were assessed by enzyme-linked immunosorbent assay (ELISA), bronchoalveolar lavage fluid (BALF) white cell count, Evans blue dye analysis, wet to dry weight ratios, and histology study. Hypoxia-inducible factor-1α (HIF-1α) siRNA and permeability assay were used to study the effect of 4-HPA on the production of inflammatory cytokines and monolayer permeability *in vitro*. The results showed that 4-HPA reduced seawater instillation-induced mortality in rats. In lung tissues, 4-HPA attenuated hypoxia, inflammation, vascular leak, and edema, and decreased HIF-1α protein level. In primary rat alveolar epithelial cells (AEC), 4-HPA decreased hypertonicity- and hypoxia-induced HIF-1α protein levels through inhibiting the activations of protein translational regulators and via promoting HIF-1α protein degradation. In addition, 4-HPA lowered inflammatory cytokines levels through suppressing hypertonicity- and hypoxia-induced HIF-1α in NR8383 macrophages. Moreover, 4-HPA decreased monolayer permeability through suppressing hypertonicity and hypoxia-induced HIF-1α, which was mediated by inhibiting vascular endothelial growth factor (*VEGF*) in rat lung microvascular endothelial cell line (RLMVEC). In conclusion, 4-HPA attenuated inflammation and edema through suppressing hypertonic and hypoxic induction of HIF-1α in seawater aspiration-induced lung injury in rats.

## 1. Introduction

Drowning, one of the three leading causes of unintentional injury death, is a major but often neglected public health problem [1]. The acute lung injury (ALI) is a serious body injuries induced by water aspiration. Some ALI induced by near-drowning would deteriorate into acute respiratory distress syndrome (ARDS) without proper treatments [2,3,4]. A recent study showed that ALI induced by seawater aspiration is severer than that by freshwater, the reason of which could concern osmotic pressure [5]. Hypoxia and hypertonicity are the two impact factors in seawater aspiration-induced ALI which had an acuter and rapider course than that induced by lipopolysaccharide (LPS) [4,6,7,8]. Besides mechanical ventilation [9,10,11], therapies which could attenuate the injuries in lungs at the early stage of seawater aspiration-induced ALI are required to prevent the occurrence of ARDS.

Hypoxia-inducible factor-1 (HIF-1) is a key transcription factor that mediates adaptive responses to changes in tissue oxygenation. It is a basic helix-loop-helix transcription factor that is composed of two subunits, HIF-1α and HIF-1β. HIF-1β is constitutively present, whereas HIF-1α protein is kept at a low or undetectable level by continuous HIF-prolyl hydroxylase domain (PHD) enzyme-mediated degradation which is suppressed by hypoxia [12]. More and more evidences have demonstrated that HIF-1α also responds to nonhypoxic stimuli such as hormones, growth factors, vasoactive peptides, cytokines, heat, LPS, and hypertonicity [13,14,15,16]. Furthermore, a previous study reported that LPS-induced lung injury could be inhibited by reducing HIF-1α expression [17]. Since both of the two impact factors, hypoxia and hypertonicity, in seawater aspiration-induced ALI affected HIF-1α which played important roles in inflammation and edema [18,19], HIF-1α could be a potential therapeutic target.

4-Hydroxyphenylacetic acid (4-HPA) is an active component of Chinese herb *Aster tataricus* (fan hun cao) which had been widely used in China for the treatment of pneumonia, HBV, and carcinoma [20,21,22]. Some previous studies reported that 4-HPA, a metabolite of aromatic amino acid catabolism that is secreted in saliva, controlled the *NadA* gene expression and could become a potential hypopigmenting agent [23,24,25]. We also found that *Aster tataricus* extract and 4-HPA could inhibit HIF-1α expression in our preliminary experiments. Therefore, we hypothesized that 4-HPA might attenuate ALI induced by seawater aspiration through inhibiting HIF-1α expression. The aim of this study is to investigate the effect of 4-HPA on seawater aspiration-induced lung injury.

## 2. Results

### 2.1. 4-Hydroxyphenylacetic Acid (4-HPA) Reduced Seawater Instillation-Induced Mortality in Rats

**Figure 1:**
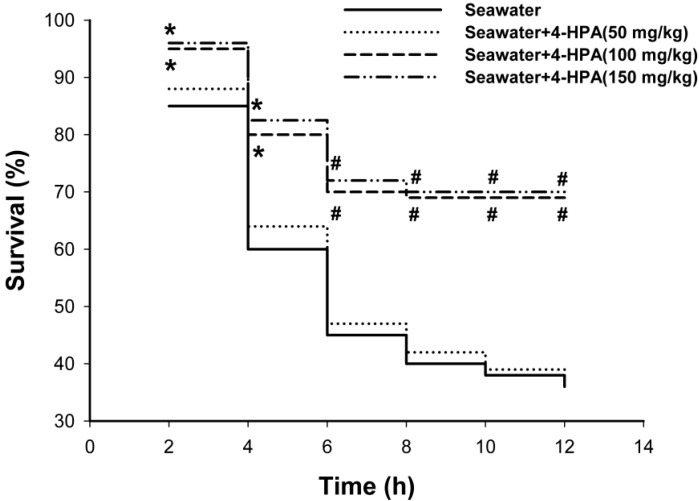
Effects of 4-hydroxyphenylacetic acid (4-HPA) on seawater instillation-induced mortality in rats. Drowning model rats were prepared with or without different does of 4-HPA (50, 100 or 150 mg/kg body weight, i.p.). 4-HPA was administered after seawater instillation for 10 min. The mortality of rats were recorded at 2, 4, 6, 8, 10, and 12 h after seawater instillation in each group (*n* = 20). * *p* < 0.05 *vs.* seawater group, ^#^ *p* < 0.01 *vs.* seawater group.

As shown in Figure 1, treatment with 4-hydroxyphenylacetic acid (4-HPA) significantly reduced seawater instillation-induced death, the accumulative mortalities during 12 h in middle dose (100 mg/kg) and high dose (150 mg/kg) of 4-HPA treatment groups were both significantly lower than that in the seawater instillation group (*p* < 0.05). However, the accumulative mortalities between middle and high does groups had no significant difference and no protection was observed when rats received 4-HPA treatment at dose of 50 mg/kg. Therefore, 100 mg/kg 4-HPA was used in the following studies.

### 2.2. 4-HPA Increased PaO_2_ and Decreased PaCO_2_ in Seawater Instillation Rats

**Figure 2:**
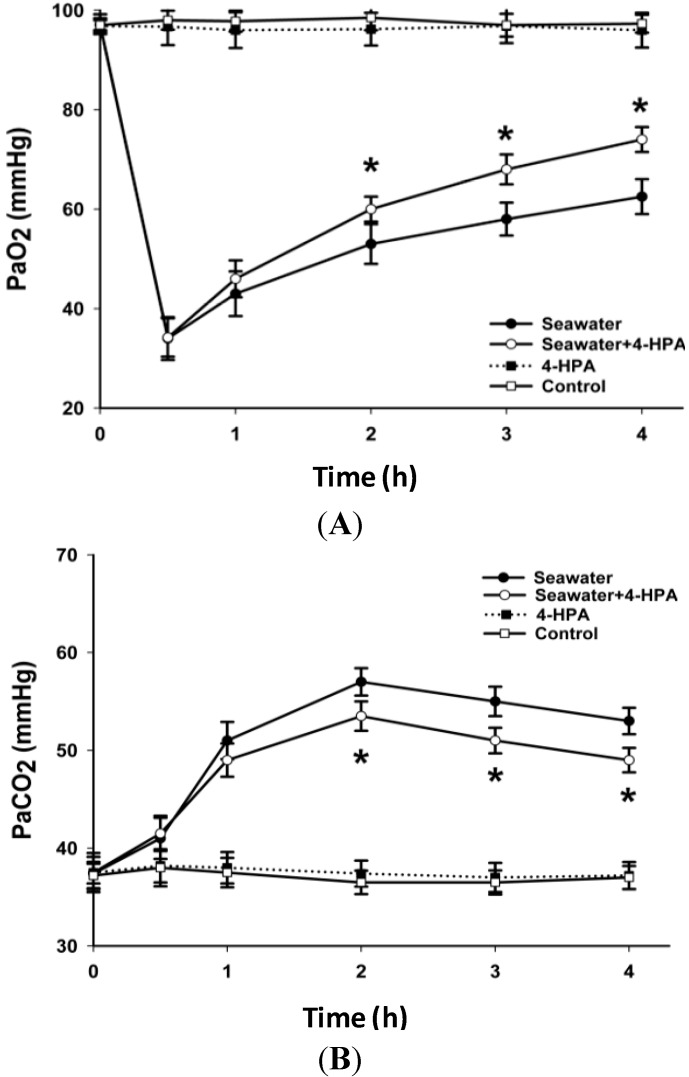
Effects of 4-HPA on PaO_2_ and PaCO_2_ after seawater instillation in rats. At 0, 0.5, 1, 2, 3, and 4 h after seawater instillation with or without 4-HPA treatment, blood samples were obtained from left carotid artery and then PaO_2_ (**A**) and PaCO_2_ (**B**) were measured by blood gas analyzer. Data are means ± standard deviation (SD) (*n* = 10), * *p* < 0.05 *vs.* seawater group.

The response of PaO_2_ and PaCO_2_ after instillation of seawater with or without treatment of 4-HPA at 0.5, 1, 2, 3, and 4 h was observed (Figure 2). The results showed that PaO_2_ dropped precipitously to its minimum at 0.5 h after instillation and then recovered gradually. The PaO_2_ of rats treated with both seawater instillation and 4-HPA were significantly higher (*p* < 0.05) than that treated with only seawater instillation at 2, 3, and 4 h. Similarly, 4-HPA decreased PaCO_2_ of rats instilled with seawater at 2, 3, and 4 h.

### 2.3. 4-HPA Attenuated Inflammation, Vascular Leak, and Edema in Seawater Instillation-Induced Lung Injury in Rats

**Figure 3:**
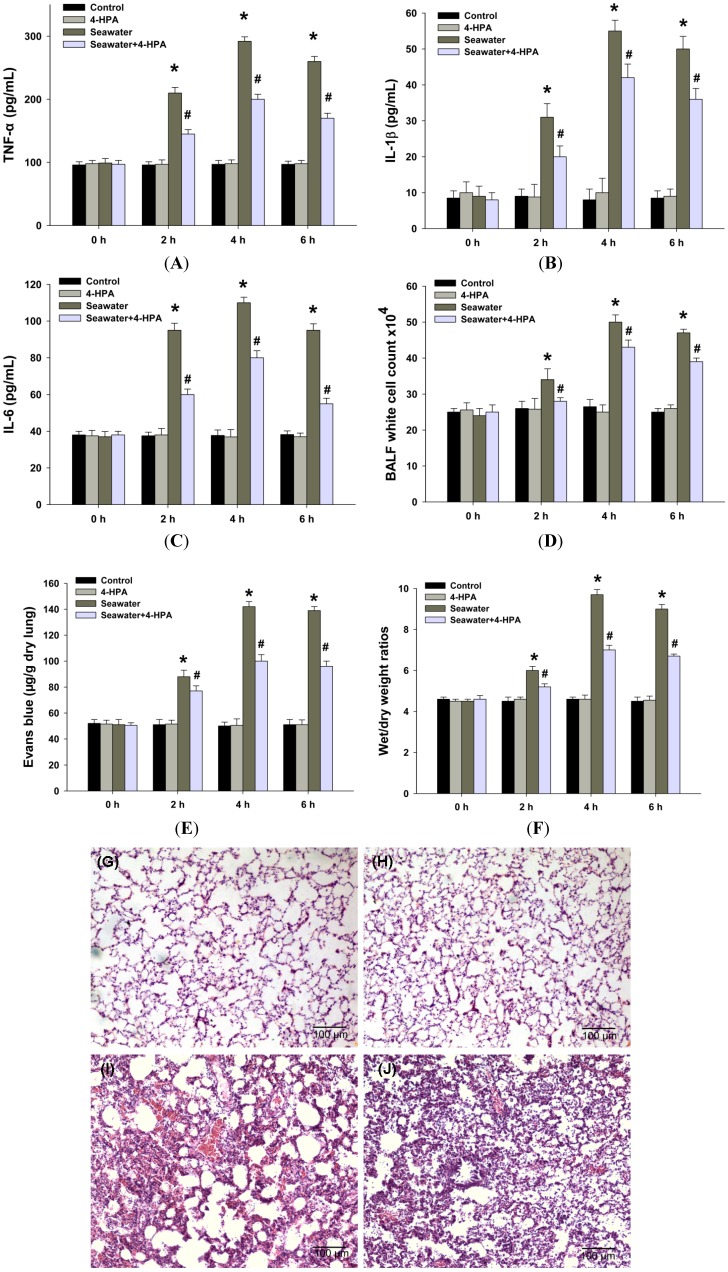
Effects of 4-HPA on inflammatory cytokines, vascular leakage, and edema after seawater instillation in lungs. After instillation of seawater for 0, 2, 4, and 6 h in the absence or presence of 4-HPA, TNF-α (**A**); IL-1β (**B**); and IL-6 (**C**) contents in lung tissues were assessed by ELISA and white cells in brochoalveolar lavage fluid (BALF) were counted (**D**); Pulmonary vascular leakage was determined by Evans blue dye analysis (**E**); Lung edema was assessed by wet to dry weight ratios (**F**); Lung tissues were stained with hematoxilin and eosin to reveal histopathological changes at 4 h after seawater aspiration. (**G**) Control group; (**H**) 4-HPA group; (**I**) Seawater group; and (**J**) Seawater + 4-HPA group. The data are presented as means ± SD from three independent experiments, * *p* < 0.05 *vs.* control group, ^#^ *p* < 0.05 *vs.* seawater group.

Inflammatory cytokines such as TNF-α, IL-1β, and IL-6 play important roles in the inflammatory response in lungs. Therefore, we detected the TNF-α (Figure 3A), IL-1β (Figure 3B), and IL-6 (Figure 3C) content to study the inflammatory response in lung tissues. After seawater instillation, the contents of TNF-α, IL-1β, and IL-6 increased at 2, 4, and 6 h (*p* < 0.05), and 4-HPA markedly inhibited the expression of these cytokines (*p* < 0.05). Additionally, the degrees of inflammation and vascular leakage in lungs were measured by bronchoalveolar lavage fluid (BALF) white cell count (Figure 3D) and Evans blue dye analysis (Figure 3E), and lung edema was assessed by wet to dry weight ratios (Figure 3F). Seawater instillation caused a significant increase in BALF white cell count, Evans blue dye analysis, and wet to dry weight ratios in seawater group compared with control (*p* < 0.05). However, administration with 4-HPA markedly reduced the three at 2, 4, and 6 h (*p* < 0.05). There was no significant difference in BALF white cell count, Evans blue dye analysis, and wet to dry weight ratios between control and 4-HPA groups in the absence of seawater instillation. The histological results showed that seawater aspiration after 4 h induced pulmonary edema, infiltration of inflammatory cells in the lung tissues and alveoli, and alveolar damage (Figure 3I). However, 4-HPA treatment could improve the lung injury (Figure 3J). There was no obvious change in the lung structure in control and 4-HPA groups (Figure 3G,H).

### 2.4. 4-HPA Decreased Seawater Instillation-Induced HIF-1α Protein Level, but not mRNA Level, in Lung Tissues in Rats

**Figure 4:**
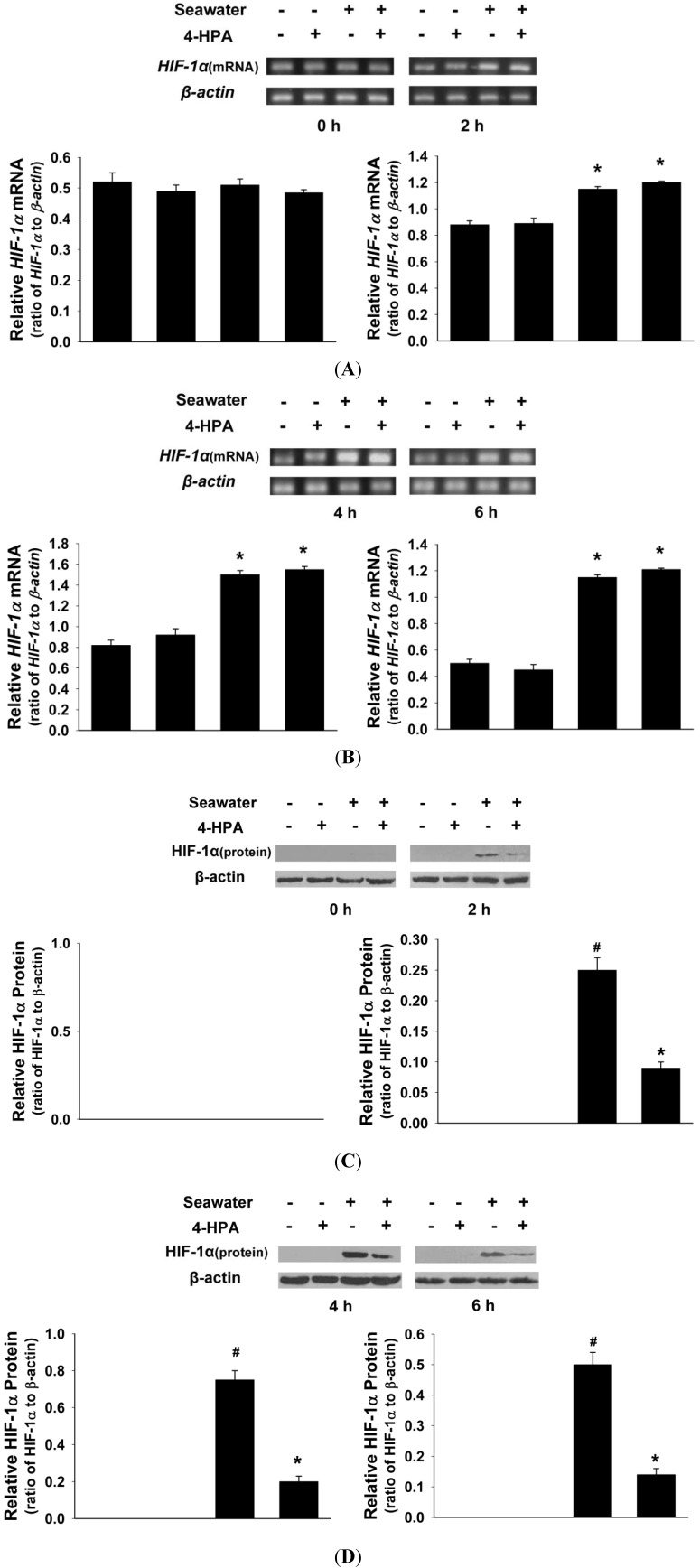
Effects of 4-HPA on seawater instillation-induced HIF-1α protein and mRNA levels in lungs. After instillation of seawater for 0, 2, 4, and 6 h in the absence or presence of 4-HPA, HIF-1α protein (**C**,**D**) and *HIF-1α* mRNA (**A**,**B**) levels of lung tissue were detected by Western blotting and RT-PCR. The ratios of HIF-1α to β-actin in protein and mRNA levels from three independent experiments were obtained by density scanning of the Western blotting and PCR band using an image analysis system. Data are means ± SD, * *p* < 0.05 *vs.* control group, ^#^ *p* < 0.05 *vs.* seawater group.

As shown in Figure 4, seawater instillation increased both HIF-1α protein and mRNA levels of lung tissue in rats at 2, 4, and 6 h (*p* < 0.05). However, 4-HPA decreased seawater instillation-induced HIF-1α protein level at each time point (*p* < 0.05), but not mRNA level. In addition, 4-HPA did not affect HIF-1α expression in the absence of seawater instillation. Since hypoxia did not affected *HIF-1α* mRNA level [26,27,28], there was hypertonicity which promoted *HIF-1α* mRNA level in seawater aspiration-induced lung injury. Therefore, there were two major injury factors and we focused on the effects of hypertonicity and hypoxia in the following studies *in vitro*. To examine that 4-HPA attenuates inflammation and edema in lung through inhibiting HIF-1α, a series of studies *in vitro* were performed as below.

### 2.5. 4-HPA Decreased Hypertonicity and Hypoxia-Induced HIF-1α Protein Level, but not mRNA Level, in Primary Rat Alveolar Epithelial Cells (AEC)

**Figure 5:**
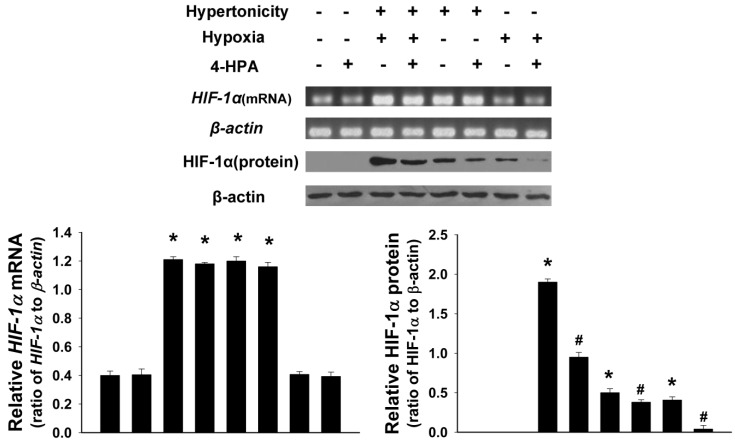
Effects of 4-HPA on hypertonicity, hypoxia, and both of the two induced HIF-1α protein and mRNA levels in primary AEC. Treated by hypertonicity, hypoxia, or both of the two with or without 4-HPA treatment for 4 h, primary rat alveolar epithelial cells (AEC) were harvested and HIF-1α protein and mRNA levels were assessed by Western blotting and RT-PCR. The ratios of HIF-1α to β-actin in protein and mRNA levels from three independent experiments were obtained by density scanning of the Western blotting and PCR band using an image analysis system. Data are means ± SD, * *p* < 0.05 *vs.* control group, ^#^ *p* < 0.05 *vs.* the groups with stimuli or stimulus in the absence of 4-HPA treatment.

As shown in Figure 5, hypertonicity (25% seawater) together with hypoxia (3% O_2_) or hypertonicity alone promoted both HIF-1α protein and mRNA expression in primary rat alveolar epithelial cells (AEC) (*p* < 0.05). Moreover, hypertonicity and hypoxia synergistically increased HIF-1α protein level. However, hypoxia alone increased only HIF-1α protein (*p* < 0.05) but not mRNA level. 4-HPA decreased hypertonicity, hypoxia, and both of the two induced HIF-1α protein levels, but not mRNA levels (*p* < 0.05).

### 2.6. 4-HPA Decreased Hypertonicity and Hypoxia-Induced HIF-1α Protein Level through Inhibiting the Activations of Protein Translational Regulators, Including p70S6K1, S6 Ribosomal Protein, 4E-BP1, and eIF4E in Primary AEC

**Figure 6:**
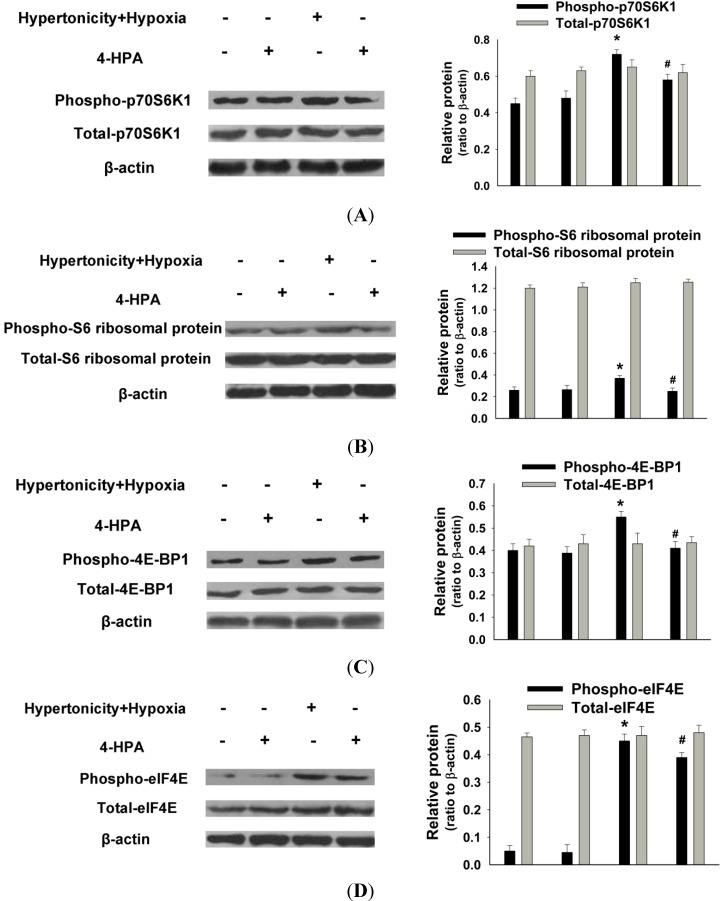
Effects of 4-HPA on HIF-1α related protein translational regulators under hypertonicity and hypoxia condition**s** in primary AEC. Primary AEC were treated by hypertonicity and hypoxia in the absence or presence of 4-HPA for 4 h. Then, the cells were harvested for Western blotting and phosphorylation and total protein levels of HIF-1α related translational regulators, including p70S6K1 (**A**); S6 ribosomal protein (**B**); 4E-BP1 (**C**); and eIF4E (**D**) were detected. The ratios of HIF-1α related translational regulators to β-actin in protein levels from three independent experiments were obtained by density scanning of the Western blotting. Data are means ± SD, * *p* < 0.05 *vs.* control group, ^#^ *p* < 0.05 *vs.* hypertonicity + hypoxia group.

Since 4-HPA did not decrease hypertonicity and hypoxia**-**induced HIF-1α protein level via the inhibition of its mRNA expression (Figure 5), posttranscriptional mechanisms were likely involved. As shown in Figure 6, hypertonicity together with hypoxia promoted the phosphorylation but not the total protein levels of HIF-1α related translational regulators, including p70S6K1, S6 ribosomal protein, 4E-BP1, and eIF4E (*p* < 0.05), which were attenuated by 4-HPA treatment (*p* < 0.05) in AEC. Additionally, 4-HPA did not affect these translational regulators under normal condition. However, the activations of these translational regulators were not promoted by either hypertonicity or hypoxia alone and not affected by 4-HPA under hypertonic or hypoxic condition (data not shown).

### 2.7. 4-HPA Decreased Hypertonicity and Hypoxia-Induced HIF-1α Protein Level through Promoting HIF-1α Protein Degradation, Which Was Associated with Prolyl Hydroxylase Domain Enzyme Isoform-2 (PHD2) Elevation in Primary AEC

**Figure 7:**
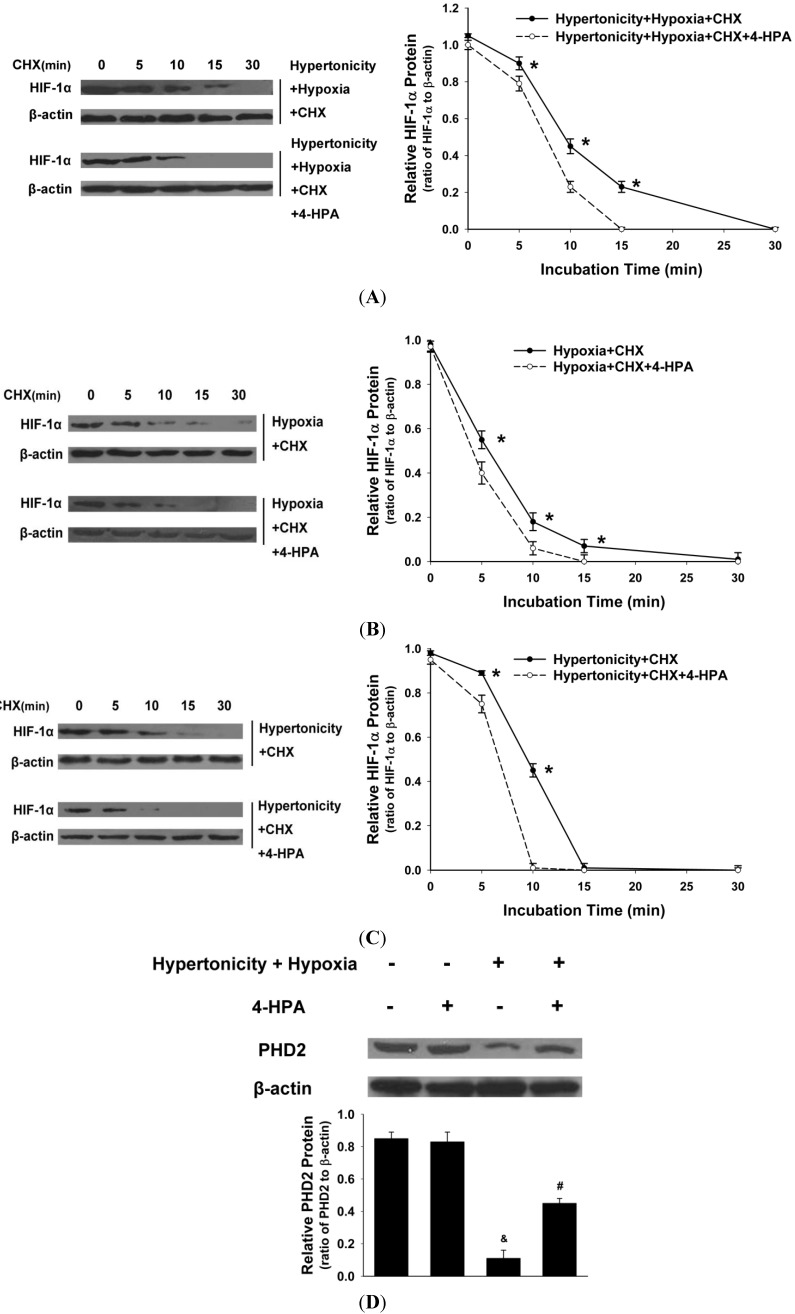
Effects of 4-HPA on HIF-1α protein degradation under hypertonicty and hypoxia conditions in primary AEC. AEC were treated with hypertonicity (**C**); hypoxia (**B**); or both of the two (**A**) with or without 4-HPA treatment for 4 h, followed by incubation with 100 μM cycloheximide (CHX, blocking ongoing protein synthesis) from 0–30 min. Cell lysates were subjected to Western blotting using antibodies against HIF-1α and β-actin (the **left** panel) and the intensity of HIF-1α protein relative content was quantified (the **right** panel). The plot represented means ± SD from three independent experiments, * *p* < 0.05 *vs.* groups with 4-HPA treatment; and (**D**) prolyl hydroxylase domain enzyme isoform-2 (PHD2) protein of AEC which were treated with both hypertonicity and hypoxia in the presence and absence of 4-HPA was detected by Western blotting. Data are means ± SD, ^&^ *p* < 0.01 *vs.* control group, ^#^ *p* < 0.05 *vs.* hypertonicity + hypoxia group.

In the presence of CHX (blocking ongoing protein synthesis), the half-life of HIF-1α protein was longer (*p* < 0.05) than that in the presence of 4-HPA treatment under hypertonic and hypoxic condition (Figure 7A) in primary AEC. Moreover, 4-HPA also increased the degradation of HIF-1α protein (*p* < 0.05) under hypoxic (Figure 7B) or hypertonic (Figure 7C) condition. As shown in Figure 7D, hypertonicity and hypoxia significantly decreased the protein level of prolyl hydroxylase domain enzyme isoform-2 (PHD2) which is an important enzyme isoform to regulate the stability of HIF-1α protein, and this effect was impaired by the treatment of 4-HPA. Our results provide the evidence that 4-HPA decreased hypertonicity and hypoxia-induced HIF-1α protein level through a mechanism that involves prolyl hydroxylase-dependent degradation.

### 2.8. 4-HPA Decreased the Production of Inflammatory Cytokines through Suppressing Hypertonicity and Hypoxia-Induced HIF-1α in NR8383 Macrophages

**Figure 8:**
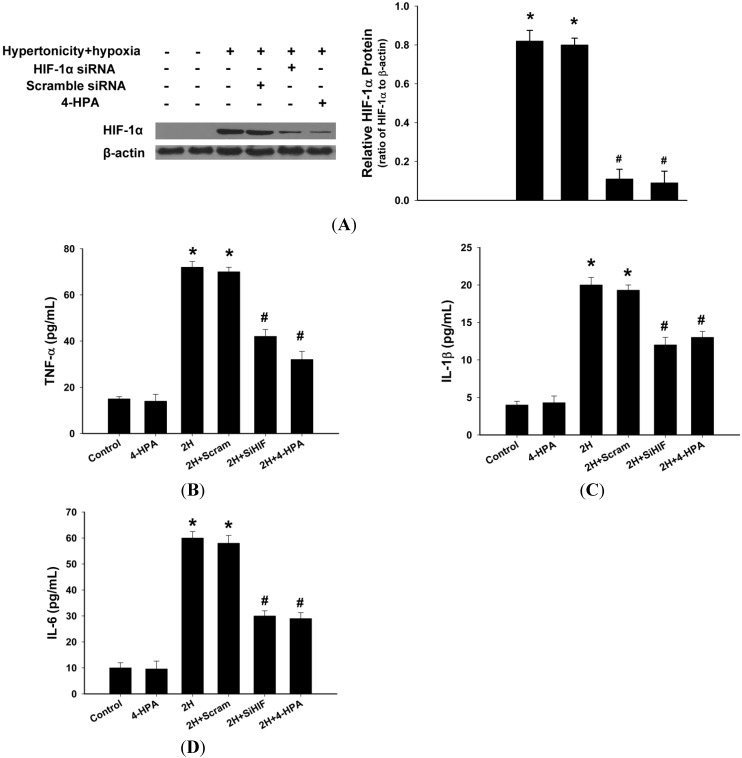
Effects of 4-HPA on inflammatory cytokines levels under hypertonicty and hypoxia conditions in NR8383 macrophages. (**A**) NR8383 cells were transfected with HIF-1α siRNA and a scramble sequence. Twenty-four hours after transfection, the cells with or without transfection were stimulated with hypertonicity and hypoxia in the absence or presence of 4-HPA for 4 h and then harvested for Western blotting; The supernatants of the cells were collected to detect the content of TNF-α (**B**); IL-1β (**C**); and IL-6 (**D**) by ELISA. Data are means ± SD from three independent experiments, * *p* < 0.05 *vs.* control group, ^#^ *p* < 0.05 *vs.* 2H group. 2H: hypertonicity and hypoxia, 2H + Scram: hypertonicity and hypoxia + scramble sequence, 2H + SiHIF: hypertonicity and hypoxia + HIF-1α siRNA.

Hypertonicity and hypoxia increased HIF-1α protein level (*p* < 0.05), which was markedly inhibited by HIF-1α siRNA or 4-HPA (*p* < 0.05) in NR8383 macrophages (Figure 8A). Meanwhile, TNF-α, IL-1β, and IL-6 contents in the supernatant from the cells stimulated with hypertonicity and hypoxia for 4 h were much more than those of control (*p* < 0.05) (Figure 8B–D). 4-HPA and HIF-1α siRNA similarly reduced the production of TNF-α, IL-1β, and IL-6. The scramble sequence exerted no significant impact on HIF-1α expression and the production of inflammatory cytokines in NR8383 macrophages.

### 2.9. 4-HPA Decreased Monolayer Permeability through Suppressing Hypertonicity and Hypoxia-Induced HIF-1α, Which Was Mediated by Inhibiting VEGF in Rat Lung Microvascular Endothelial Cell Line (RLMVEC)

**Figure 9:**
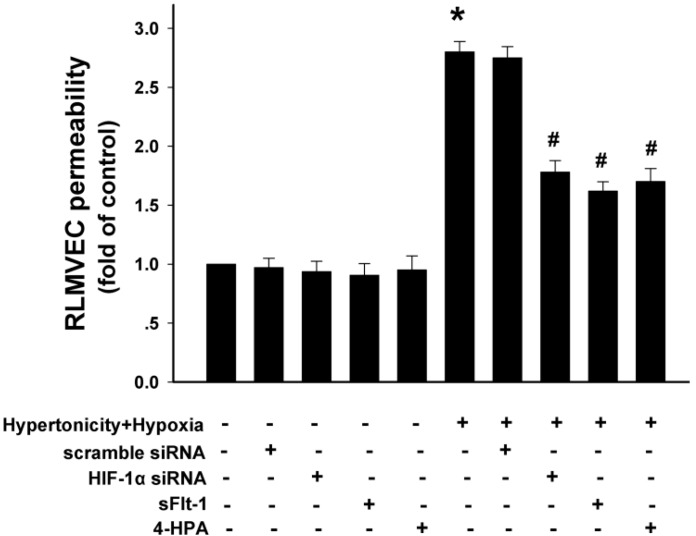
Effects of 4-HPA on monolayer permeability under hypertonicity and hypoxia conditions in rat lung microvascular endothelial cells (RLMVEC). RLMVEC were stimulated with HIF-1α siRNA, sFlt-1 (1 μM, vascular endothelial growth factor (*VEGF*)antagonist), 4-HPA, hypertonicity and hypoxia, hypertonicity and hypoxia + HIF-1α siRNA, hypertonicity and hypoxia + sFlt-1, hypertonicity and hypoxia + 4-HPA for 4 h, respectively. The RLMVEC monolayer permeability was measured with FITC-albumin (50 μM). Data are means ± SD, * *p* < 0.01 *vs.* control group, ^#^ *p* < 0.05 *vs.* hypertonicity and hypoxia group.

As shown in Figure 9, hypertonicity and hypoxia increased rat lung microvascular endothelial cell line (RLMVEC) monolayer permeability (*p* < 0.01), and HIF-1α siRNA, sFlt-1 (*VEGF* antagonist), or 4-HPA did not affected it significantly during normal culture condition. Furthermore, hypertonicity and hypoxia-induced elevation of RLMVEC monolayer permeability was suppressed by HIF-1α siRNA, sFlt-1, and 4-HPA, respectively (*p* < 0.05) and there was no significant difference between these suppression effects. *VEGF* is a standard target gene of *HIF-1*. Therefore, 4-HPA decreased monolayer permeability through suppressing hypertonicity and hypoxia-induced HIF-1α, which was mediated by inhibiting *VEGF* in RLMVEC.

## 3. Discussion

The acute lung injury (ALI) induced by seawater aspiration had an acuter and rapider course than that induced by LPS [4,6,7,8]. The majority death induced by seawater aspiration happened within the first 6 h (Figure 2), which is greatly shorter than 40 h in LPS-induced ALI [29]. As Figure 2 and Figure 3 shown, PaO_2_ dropped precipitously to its minimum at 0.5 h and inflammation and edema in lungs were most serious at 4 h after seawater instillation in rats. These time points were also earlier than those of some other ALI [30,31]. Since the results (Figure 1, Figure 2 and Figure 3) suggested that 4-HPA could attenuate the injuries in lungs rapidly after treatment, the agent should be used in the early stage of seawater aspiration-induced ALI.

The high level of PaCO_2_ after seawater instillation suggested that there was pulmonary ventilatory insufficiency and local lung hypoxia. The treatment of 4-HPA increased PaO_2_ and decreased PaCO_2_ in seawater instillation rats (Figure 2). Since PaO_2_ and PaCO_2_ could indicate, at least partially, the degree of hypoxia within local lung tissue, 4-HPA might attenuate hypoxia in lung tissues in rats, which in turn inhibited HIF-1α indirectly. On the one hand, 4-HPA attenuated inflammation and edema within lung tissues to improve pulmonary membrane oxygenation, which could help to increase PaO_2_. On the other hand, the Chinese traditional medicine used *Aster tataricus* of which 4-HPA is an active component to reduce and expel phlegm. Therefore, 4-HPA might have a similar function and decrease PaCO_2_ through improving obstructive ventilatory disorder, which was not investigated in the current study.

Hypoxia increases HIF-1α protein by elevating its protein stability but not by prompting its mRNA expression [4,6,7,8]. However, instillation of seawater increased not only HIF-1α protein but also its mRNA expression (Figure 4). Therefore, hypertonicity induced by seawater instillation could be the factor which was accounted for the increase of *HIF-1α* mRNA expression. This hypothesis was demonstrated by our following experiments *in vitro* (Figure 5). Some previous studies reported that HIF-1α also responded to nonhypoxic stimuli such as hormones, growth factors, vasoactive peptides, cytokines, heat, hypertonicity, and LPS [13,14,15,16]. Our study demonstrated again that hypertonicity was a nonhypoxic stimulus of HIF-1α (Figure 4 and Figure 5). In addition, a previous study reported that LPS induced HIF-1α synergistically with hypoxia [32]. Our results showed that hypertonicity could synergize with hypoxia to increase HIF-1α protein (Figure 5). Since our results showed that hypertonicity and hypoxia synergistically increased only HIF-1α protein but not mRNA level, posttranscriptional mechanisms were likely involved in this synergistic effect. We found hypertonicity together with hypoxia promoted the phosphorylation but not the total protein levels of HIF-1α related translational regulators, including p70S6K1, S6 ribosomal protein, 4E-BP1, and eIF4E, which were attenuated by 4-HPA treatment in AEC (Figure 6). 4-HPA inhibited the synergistic effect of hypertonicity and hypoxia on HIF-1α probably through suppressing synergistic protein translational process, which need further study to demonstrate. Although HIF-1α related protein translational regulators were not affected by hypertonicity or hypoxia alone (data not shown), HIF-1α protein degradation was suppressed by hypertonicity or hypoxia alone, which could be inhibited by 4-HPA (Figure 7). The HIF-1α protein levels which can be detected by Western blotting are mainly decided by three processes which are the transcription, translation, and degradation of HIF-1α protein. 4-HPA impacted HIF-1α protein levels following either hypertonicity or hypoxia through promoting HIF-1α protein degradation (Figure 7B,C) but not through impacting these translational regulators (data not shown). Moreover, 4-HPA decreased HIF-1α protein levels following both hypertonicity and hypoxia through both inhibiting the activations of protein translational regulators (Figure 6) and promoting HIF-1α protein degradation (Figure 7A). In addition, hypertonicity or hypoxia alone did not impact these translational regulators but hypertonicity together with hypoxia synergistically increased them. Therefore, this synergistic effects might be impacted by 4-HPA which was not investigated in the current study. Taken all together, there are hypertonic and hypoxic factors in seawater aspiration-induced lung injury and these two main impact factors synergistically increased HIF-1α protein which was attenuated by 4-HPA. Therefore, our study focused on how the effects of the two factors together *in vitro* were affected by 4-HPA.

HIF-1 is a transcription factor which is essential for regulating oxygen homeostasis. It also regulates the expression of target genes important in angiogenesis, erythropoiesis, energy metabolism, and cell survival. However, there would be more detrimental effects of HIF-1α than beneficial ones when the injury factor such as LPS leads to HIF-1α overexpression, which needs an agent to inhibit [17]. In our study, the greatly increased HIF-1α protein induced synergistically by hypertonic and hypoxic factors contributed to the seawater aspiration-induced lung injury and we found that 4-HPA was a proper agent to attenuate the ALI by inhibiting HIF-1α.

4-HPA which can be secreted in saliva is a metabolite of aromatic amino acid catabolism. It is also an active component of some Chinese herb such as *Aster tataricus* and *Rhodiola rosea*, as well as an intermediate product of chemical synthesis of atenolol (β-receptor blocking agent) and puerarin (active component of *Lobed Kudzuvine Root*). Some previous studies reported that 4-HPA controlled the *NadA* gene expression and could become a potential hypopigmenting agent [23,24,25]. Our results suggested that 4-HPA was a good inhibitor of hypertonicity, hypoxia, or both of the two-induced HIF-1α expression. Additionally, 4-HPA, a water-soluble small molecular compound, could be a potential agent for the diseases with hypoxia-induced inflammation or edema and some carcinomas of which hypoxia-induced HIF-1α promoted the progress [33]. Moreover, HIF-1α played important roles in some diseases under hypertonic condition, such as diabetic eye disease [34] and some renal diseases [35]. Therefore, 4-HPA might be used in these fields.

## 4. Experimental Section

### 4.1. Animal Model and Grouping

Male Sprague–Dawley rats, weighing 180–220 g each, were obtained from the Animal Center (Fourth Military Medical University, Xi’an, China). These rats were kept in a temperature-controlled house with 12 h light-dark cycles and fed with standard laboratory diet and water *ad libitum*. All experiments approved by the Animal Care and Use Committee of the Fourth Military Medical University conformed to the Declaration of the National Institutes of Health Guide for Care and Use of Laboratory Animals (Publication No.85–23, revised in 1985).

Seawater Drowning Animal Model: the rats were anesthetized with 3% sodium pentobarbital (1.5 mL/kg, Sigma-Aldrich, St. Louis, MO, USA) intraperitoneally and maintained in the supine position during experiments with the head elevated 30°. A catheter was inserted into the right jugular artery to obtain blood samples. A 1 mL syringe was gently inserted into the trachea approximately 1.5 cm above the carina. Then, 4 mL/kg body weight of seawater was instilled within 4 min into both lungs. Seawater (osmolality 1300 mOsm/kgH_2_O, pH 8.2, temperature 25 °C, specific weight (SW) 1.05, NaCl 26.518 g/L, MgSO_4_ 3.305 g/L, MgCl_2_ 2.447 g/L, CaCl_2_ 1.141 g/L, KCl 0.725 g/L, NaHCO_3_ 0.202 g/L, NaBr 0.083 g/L) was prepared according to the major composition of the East China Sea provided by Chinese Ocean Bureau (Beijing, China).

**Figure 10:**
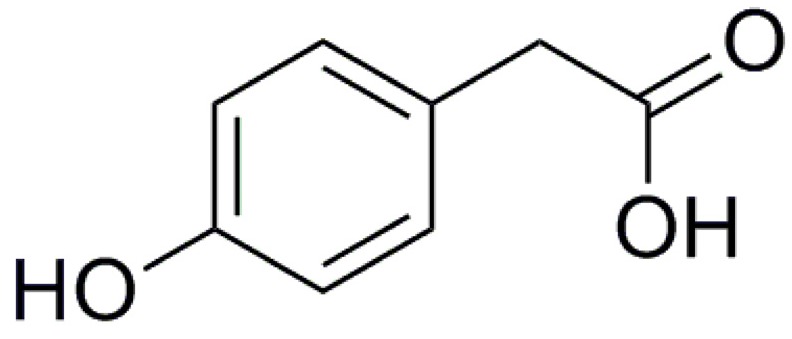
The chemical structure of 4-HPA.

To assess mortality of rates, drowning rat models were prepared as mentioned before with or without different does of 4-HPA (50, 100 or 150 mg/kg body weight, i.p.) (solubility of 4-HPA, 150 mg/mL, pH 7.4). 4-HPA (Sigma-Aldrich, St. Louis, MO, USA, the chemical structure is showed in Figure 10) was administered after seawater instillation for 10 min. The mortality of rats were recorded every 2 h after seawater instillation in each group (*n* = 20).

Rats were randomly divided into four groups (*n* = 40), (1) control; (2) 4-HPA (100 mg/kg) only; (3) seawater only; and (4) seawater + 4-HPA (100 mg/kg). In the seawater + 4-HPA group, rats were treated with 100 mg/kg 4-HPA after seawater instillation for 15 min. Rats were sacrificed at 2, 4, or 6 h after seawater instillation. Then, the thorax was opened rapidly and lungs were processed for studies in the manner described below.

### 4.2. PaO_2_ and PaCO_2_ Study

In each group, blood samples were obtained from a PE-50 catheter which was inserted in the right carotid artery at 0, 1, 2, 3, or 4 h after seawater instillation. Then, arterial oxygen tension (PaO_2_) and arterial carbon dioxide tension (PaCO_2_) of the blood samples was measured with a blood gas analyzer (Ymb-3100, Yima Opto-Electrical Technology Co., Ltd., Xi’an, China).

### 4.3. ELISA

The concentrations of TNF-α, IL-1β, and IL-6 were measured by using ELISA kits (R&D systems; Minneapolis, MN, USA). Lungs tissue and cells supernatant were processed according to the manufacturer’s instructions for ELISA.

### 4.4. Bronchoalveolar Lavage Fluid (BALF) White Cell Count

After seawater instillation for 0, 2, 4, or 6 h, rats were anesthetized with intraperitoneal pentobarbital. The lungs were lavaged with 2.5 mL ice-cold phosphate buffered saline five times in all groups. The recovery ratio of the fluid was about 90%. The collected bronchoalveolar lavage fluid (BALF) was centrifuged at 520× *g* for 20 min at 4 °C. The cell pellet was then resuspended in 1 mL of red blood cell-lysis buffer to eliminate red cells. White cells were then re-pelleted by centrifugation at 520× *g* for 20 min at 4 °C. The cell pellet was again resuspended in phosphate buffer saline (PBS) and taken for cell counting using a hemocytometer.

### 4.5. Assessment of Pulmonary Vascular Leakage

Pulmonary vascular leakage was assessed as previously described [2] by quantitating extravasation of Evans blue into lung parenchyma. In brief, Evans blue dye (20 mg/kg, Sigma-Aldrich, St. Louis, MO, USA) was given intravenously to the rats 30 min before the animals were sacrificed. After the lung was rapidly removed from the thoracic cavity, normal saline was immediately injected into the right ventricle till there was effused clear fluid from the left atrium. The lung was removed and dried to a constant weight at 60 °C for 72 h. The dried lung was weighed and then incubated in formamide (3 mL/100 mg, Sigma-Aldrich, St. Louis, MO, USA) at 60 °C for 24 h. Then, the supernatant was separated by centrifugation at 5000× *g* for 30 min. The concentration of Evans blue in the supernatant was quantitated by the following formula: A620 (correction) = A620 − (1.426 × A740 + 0.030). The concentration of Evans blue in the lung tissue (ET) was determined from the generated Evans blue standard absorbance curves. At the same time, the concentration of Evans blue in blood (EB) was detected by the same method as described above. ELI = ET/(EB × dry lung weight) × 100%.

### 4.6. Wet-to-Dry Weight (W/D) Ratio

Wet-to-dry weight (W/D) ratio was used to represent the severity of lung edema. After lungs were separated from the thoracic cavity at the end of the experiment, the left lungs were weighed and then dried to constant weight at 50 °C for 72 h. The ratio of wet-to-dry was finally calculated by dividing the wet weight by the dry weight.

### 4.7. Histological Study

At the end of the experiments, the lung tissues were fixed with 4% paraformaldehyde for 24 h and embedded in paraffin. After deparaffinisation and dehydration, the lungs were cut into 5 μm sections and stained with hematoxylin and eosin.

### 4.8. Cell Culture and Treatment

Cell isolation and preparation of primary AEC (rat alveolar epithelial cells) monolayers: AT2 (alveolar epithelial type II) cells were isolated from adult male Sprague-Dawley rats (125–150 g) by disaggregation with elastate (2.0–2.5 U/mL; Worthington Biochemical, Freehold, NJ, USA), followed by differential adherence on IgG-coated bacteriological plates. All animals were treated in accordance with the guidelines and approval of the Fourth Military Medical University Institutional Animal Care and Use Committee. Freshly isolated AT2 cells were plated in minimal defined serum-free medium (MDSF) consisting of Dulbecco’s modified Eagle’s medium and Ham’s F-12 nutrient mixture in a 1:1 ratio, supplemented with 1.25 mg/mL bovine serum albumin, 10 mM HEPES, 0.1 mM nonessential amino acids, 2.0 mM glutamine, 100 U/mL sodium penicillin G, and 100 μg/mL streptomycin. Cells were seeded onto tissue culture-treated polycarbonate filter cups (Nuclepore, Corning-Costar, Corning, NY, USA, 0.4 μm) at a density of 1.0 × 10^6^ cells/cm^2^ and grown to confluence for RNA and protein analyses. Media were changed on the second day after plating and every other day thereafter. Cultures were maintained in a humidified 5% CO_2_ incubator at 37 °C. AT2 cell purity (>90%) was assessed by staining freshly isolated cells with tannic acid or an antibody (Ab) to a lamellar membrane protein, p180 (Covance Research, Berkeley, CA, USA), followed by immunofluorescence visualization. Cell viability (>95%) was measured by trypan blue dye exclusion. Day of isolation is designated as day 0; cells were used on day 3 or 4.

Hypertonicity *in vitro*: Hypertonicity was induced by 25% seawater (seawater volume/(seawater volume + Ham’s F12 medium volume) = 25%), osmolality 538.21 ± 0.99 mOsm/kgH_2_O, pH 8.2, temperature 25 °C. In our preliminary experiment, HIF-1α protein level reached its maximum when AEC were stimulated by 25% seawater, compared with 12.5% or 37.5% seawater.

Hypoxia *in vitro*: AEC were cultured in a special humidified hypoxic chamber as previously described [19]. Use of an antechamber ensured that once cells became hypoxic, they were never re-exposed to a normoxic environment. The chamber utilized a positive pressure system and was supplied with a gas mixture of 3% O_2_, 5% CO_2_, and the balance nitrogen. Culture media and seawater (25%) used in the test were allowed to equilibrate to 3% O_2_ before initiation of the test.

For effects of 4-HPA on HIF-1α expression induced by hypertonicity, hypoxia, or both of the two, primary AEC cells were stimulated with hypertonicity, hypoxia, or both of the two in the presence or absence of 4-HPA (100 μg/mL, Sigma-Aldrich, St. Louis, MO, USA) for 4 h. After being stimulated by diverse stimulus, HIF-1α protein, mRNA, HIF-1α related protein translational regulators (p70S6K1, S6 ribosomal protein, 4E-BP1, eIF4E), and HIF-prolyl hydroxylase domain enzyme isoform-2 (PHD2) protein levels of the cells were detected with Western blotting and RT-PCR. Additionally, stimulated AEC were incubated with 100 μM cycloheximide (CHX, blocking ongoing protein synthesis, obtained from EMD Biosciences, San Diego, CA, USA) from 0–30 min, then the cells were harvested at indicated time points for Western blotting.

### 4.9. Plasmid Construction and Transfection

The rat alveolar macrophage cell line NR8383 (endowed by Pharmacology Department, Fourth Military Medical University, Xi’an, China) was maintained in Ham’s F12 medium (Sigma-Aldrich, St. Louis, MO, USA) supplemented with 10% fetal calf serum (FCS) (Sigma-Aldrich, St. Louis, MO, USA), 100 U/mL of penicillin (Sigma-Aldrich, St. Louis, MO, USA) and 100 μg/mL of streptomycin (Sigma-Aldrich, St. Louis, MO, USA) at 37 °C in a humidified atmosphere containing 5% CO_2_ and 95% air.

HIF-1α siRNA was used to study the effect of 4-HPA on the production of inflammatory cytokines during hypertonic and hypoxic condition. The HIF-1α siRNA and the scramble sequence (Invitrogen, Grand Island, NY, USA) were kindly provided by Lili Liu (Department of Oncology, Tangdu Hospital, Fourth Military Medical University, Xi’an 710038, China), and the siRNA expression vector for HIF-1α was constructed as described previously [36]. NR8383 macrophages and rat lung microvascular endothelial cell line (RLMVEC) were transfected with HIF-1α siRNA or a scramble sequence by using Lipofectamine 2000 (Invitrogen, Carlsbad, CA, USA) according to the manufacturer’s instructions. Twenty-four hours after transfection, NR8383 cells with or without transfection were stimulated with both of hypertonicity and hypoxia for 4 h and harvested for Western blotting to evaluate the changes of HIF-1α expression. Meanwhile, the supernatant was collected to detect the contents of TNF-α, IL-1β, and IL-6 by ELISA. In addition, RLMVEC with or without transfection were used in permeability assay below.

### 4.10. Permeability Assay

Rat lung microvascular endothelial cells (RLMVEC, VEC Technologies, Rensselaer, NY, USA) were cultured in high-glucose DMEM (Sigma-Aldrich, St. Louis, MO, USA) supplemented with 5% fetal bovine serum (FCS) (Sigma-Aldrich, St. Louis, MO, USA), 50 U/mL of penicillin (Sigma-Aldrich, St. Louis, MO, USA) and 50 μg/mL of streptomycin (Sigma-Aldrich, St. Louis, MO, USA) at 37 °C in a humidified atmosphere containing 5% CO_2_ and 95% air. RLMVEC were seeded (~100,000 cells/insert) on polystyrene filters (No.3470, 6.5-mm diameter, 0.4-μm pore size; Costar Transwell, Cambridge, MA, USA). RLMVEC were grown to confluence over 48 h after which the cells were treated with (1) standard cell culture conditions; (2) HIF-1α siRNA; (3) 1 μM soluble VEGF receptor-1 (sFlt-1, provided by professor Lan Yang, No. 210 Hospital of PLA, Dalian, China); (4) 4-HPA (100 µg/mL); (5) hypertonicity + hypoxia; (6) hypertonicity + hypoxia + HIF-1α siRNA; (7) hypertonicity + hypoxia + sFlt-1; and (8) hypertonicity + hypoxia + 4-HPA for 4 h, respectively. Then, the RLMVEC were washed three times in serum-free medium, and FITC-labeled albumin (50 μM, Sigma-Aldrich, St. Louis, MO, USA) suspended in serum-free medium was added to the RLMVEC monolayers (100 μL, VEC Technologies, Rensselaer, NY, USA). The insert was placed in a new well of a 24-well plate containing serum-free medium (0.6 mL to ensure that the fluid volume on either side of the inserts was equalized to avoid a hydrostatic gradient that might alter the rate of albumin flux). Measuring the increase of fluoresceinisothiocyanate (FITC)-albumin in the lower well after 1 h assessed the transfer rate of albumin across the monolayer. FITC-albumin was quantified in a F4500 fluorimeter (Hitachi, Tokyo, Japan) and compared with a standard curve of fluorescence made with various dilutions of the FITC-albumin. The measurements of permeability were expressed as the fold of control.

### 4.11. Western Blotting

The lung tissues and cultured cells were prepared to extract proteins with lysis buffer (10 mM Tris pH 8.0, 1 mM EDTA, 400 mM NaCl, 10% glycerol, 0.5% NP-40, 5 mM sodium fluoride, 0.1 mM phenylmethylsulfonyl fluoride, 1 mM dithiothreitol). The lysates were centrifuged at 12,000 rpm for 30 min at 4 °C, and then supernatants were collected. Equal amounts (50 μg) of protein were separated by SDS-PAGE, transferred to nitrocellulose membrane at 100 V for 2.5 h at low temperature, and blocked with 5% skim milk for 2 h. Subsequently, anti-HIF-1α antibody (dilution 1:1000; Millipore, Bedford, MA, USA), anti-p70S6K1 (*M*r 70,000 ribosomal protein S6 kinase 1, 1:1000; Cell Signaling Technology, Danvers, MA, USA), anti-Phospho-p70S6K1 (Phosphorylated Thr-421/Ser-424) (1:500; Cell Signaling Technology, Danvers, MA, USA), anti-S6 ribosomal protein (1:100; Cell Signaling Technology, Danvers, MA, USA), anti-Phospho-S6 ribosomal protein (Ser-234/236) (1:500; Cell Signaling Technology, Danvers, MA, USA), anti-4E-BP1 (eukaryotic initiation factor 4E (eIF4E)-binding protein 1, 1:500, Cell Signaling Technology, Danvers, MA, USA), anti-Phospho-4E-BP1 (Ser-65) (1:500; Cell Signaling Technology, Danvers, MA, USA), anti-eIF4E (1:1000; Cell Signaling Technology, Danvers, MA, USA), anti-Phospho-eIF4E (Ser-209) (1:1000; Cell Signaling Technology), anti-PHD2 (1:1000, Sigma-Aldrich, St. Louis, MO, USA), and anti-β-actin (1:2000, Sigma-Aldrich, St. Louis, MO, USA) were respectively added and kept with the membranes at 4 °C overnight. After repeated washing, the membranes were incubated with horseradish peroxidase-conjugated anti-rabbit secondary antibody (1:2000, Sigma-Aldrich, St. Louis, MO, USA) and bands visualized by using the enhanced chemiluminescence (ECL) system (Amersham Pharmacia Biotech, Arlington Heights, IL, USA). The results were expressed as the ratio to β-actin level in the same protein samples.

### 4.12. Reverse Transcription-PCR

Total RNA was extracted respectively from lung tissues and cells with Trizol (Invitrogen, Grand Island, NY, USA) according to the manufacturer’s instructions. The total RNA concentration was determined by spectrometric analysis. *HIF-1α* mRNA was examined by reverse transcription-PCR (RT-PCR) as per the manufacturer’s instructions (Promeaga, Madison, WI, USA). A total of 1 μg of RNA was incubated with reverse transcriptase mixture at 50 °C for 30 min, followed by amplification with the specific primers (synthesized by Invitrogen). The primers for *HIF-1α* (575 bp) were (forward) 5'-GACACCGCGGGCACCGATT-3', and (reverse) 5'-GTTCATCGTCCTCCCCCGGC-3', and for *β-actin* (240 bp), were (forward) 5'-TAAAGACCTCTATGCCAACACAGT-3', and (reverse) 5'-CACGATGGAGGGCCGGACTCATC-3', respectively. Thirty-five amplification cycles consisting of 30 s of denaturation at 95 °C, 30 s of annealing at 55 °C, and 1 min of extension at 72 °C were performed. After amplification, the RT-PCR products were separated on 1% agarose gels (Invitrogen, Grand Island, NY, USA), and the bands were visualized by ethidium bromide (Invitrogen, Grand Island, NY, USA) staining. Quantitation was obtained by density scanning of the PCR bands using a Scion image analysis system (Beta 4.02, Scion Corporation, Colombo, Sri Lanka). The results were expressed as the ratio to β-actin mRNA level in the same RNA samples.

### 4.13. Statistical Analysis

Data are expressed as means ± SD Statistically significant differences between groups were determined by ANOVA followed by Student’s test. Survival data were presented by the Kaplan Meier method [37] and comparisons were made by the log rank test. A statistical difference was accepted as significant if *p <* 0.05.

## 5. Conclusions

4-HPA attenuated inflammation and edema through the suppression of hypertonic and hypoxic induction of HIF-1α in seawater aspiration-induced lung injury in rats. It may be considered as a potential agent in treatment of seawater aspiration-induced lung injury.
